# Race/Ethnic Differences in the Associations of the Framingham Risk Factors with Carotid IMT and Cardiovascular Events

**DOI:** 10.1371/journal.pone.0132321

**Published:** 2015-07-02

**Authors:** Crystel M. Gijsberts, Karlijn A. Groenewegen, Imo E. Hoefer, Marinus J. C. Eijkemans, Folkert W. Asselbergs, Todd J. Anderson, Annie R. Britton, Jacqueline M. Dekker, Gunnar Engström, Greg W. Evans, Jacqueline de Graaf, Diederick E. Grobbee, Bo Hedblad, Suzanne Holewijn, Ai Ikeda, Kazuo Kitagawa, Akihiko Kitamura, Dominique P. V. de Kleijn, Eva M. Lonn, Matthias W. Lorenz, Ellisiv B. Mathiesen, Giel Nijpels, Shuhei Okazaki, Daniel H. O’Leary, Gerard Pasterkamp, Sanne A. E. Peters, Joseph F. Polak, Jacqueline F. Price, Christine Robertson, Christopher M. Rembold, Maria Rosvall, Tatjana Rundek, Jukka T. Salonen, Matthias Sitzer, Coen D. A. Stehouwer, Michiel L. Bots, Hester M. den Ruijter

**Affiliations:** 1 Department of Experimental Cardiology, University Medical Center Utrecht, Utrecht, the Netherlands; 2 Interuniversity Cardiology Institute of the Netherlands, Utrecht, The Netherlands; 3 Julius Center for Health Sciences and Primary Care, University Medical Center Utrecht, Utrecht, the Netherlands; 4 Department of Cardiology, Division Heart and Lungs, University Medical Centre Utrecht, Utrecht, The Netherlands; 5 Durrer Center for Cardiogenetic Research, ICIN-Netherlands Heart Institute, Utrecht, The Netherlands; 6 Institute of Cardiovascular Science, faculty of Population Health Sciences, University College London, London, United Kingdom; 7 Department of Cardiac Sciences and Libin Cardiovascular Institute of Alberta, University of Calgary, Alberta, Canada; 8 Department of Epidemiology and Public Health University College London, London, United Kingdom; 9 Institute for Health and Care Research, VU University Medical Center, Amsterdam, The Netherlands; 10 Dept of Clinical Sciences in Malmö, Lund University, Skåne University Hospital, Malmö, Sweden; 11 Department of Biostatistical Sciences and Neurology, Wake Forest School of Medicine, Winston-Salem, NC, United States of America; 12 Department of General Internal Medicine, Division of Vascular Medicine, Radboud University Nijmegen Medical Centre, Nijmegen, the Netherlands; 13 University of Malaya Medical Center, Kuala Lumpur, Malaysia; 14 Dept of Clinical Sciences in Malmö, Lund University, Skåne University Hospital, Malmö, Sweden; 15 Department of General Internal Medicine, Division of Vascular Medicine, Radboud University Nijmegen Medical Centre, Nijmegen, the Netherlands; 16 Osaka Medical Center for Health Science and Promotion, Osaka, Japan; 17 Department of Neurology, Tokyo Women Medical University, Tokyo, Japan; 18 Cardiovascular Research Institute & Surgery, Singapore, Singapore; 19 Department of Medicine, Division of Cardiology and Population Health Research Institute, McMaster University, Hamilton, Ontario, Canada; 20 Department of Neurology, University Hospital, Goethe-University, Frankfurt am Main, Germany; 21 Brain and Circulation Research Group, Department of Clinical Medicine, University of Tromsø, Tromsø, Norway; 22 Institute for Health and Care Research, VU University Medical Center, Amsterdam, The Netherlands; 23 Stroke Center, Department of Neurology, Osaka University Graduate School of Medicine, Osaka, Japan; 24 Department of Radiology, Tufts Medical Center, Boston, MA, United States of America; 25 Centre for Population Health Sciences, University of Edinburgh, Edinburgh, United Kingdom; 26 Cardiology Division, Department of Internal Medicine, University of Virginia, Charlottesville, VA, United States of America; 27 Department of Neurology, Miller School of Medicine, University of Miami, Miami, FL, United States of America; 28 MAS-Metabolic Analytical Services Oy, Helsinki, Finland; 29 Department of Neurology, University Hospital, Goethe-University, Frankfurt am Main, Germany and Department of Neurology Klinikum Herford, Germany; 30 Department of Internal Medicine and Cardiovascular Research Institute Maastricht, Maastricht University Medical Center, Maastricht, The Netherlands; University of Pittsburgh Center for Vaccine Research, UNITED STATES

## Abstract

**Background:**

Clinical manifestations and outcomes of atherosclerotic disease differ between ethnic groups. In addition, the prevalence of risk factors is substantially different. Primary prevention programs are based on data derived from almost exclusively White people. We investigated how race/ethnic differences modify the associations of established risk factors with atherosclerosis and cardiovascular events.

**Methods:**

We used data from an ongoing individual participant meta-analysis involving 17 population-based cohorts worldwide. We selected 60,211 participants without cardiovascular disease at baseline with available data on ethnicity (White, Black, Asian or Hispanic). We generated a multivariable linear regression model containing risk factors and ethnicity predicting mean common carotid intima-media thickness (CIMT) and a multivariable Cox regression model predicting myocardial infarction or stroke. For each risk factor we assessed how the association with the preclinical and clinical measures of cardiovascular atherosclerotic disease was affected by ethnicity.

**Results:**

Ethnicity appeared to significantly modify the associations between risk factors and CIMT and cardiovascular events. The association between age and CIMT was weaker in Blacks and Hispanics. Systolic blood pressure associated more strongly with CIMT in Asians. HDL cholesterol and smoking associated less with CIMT in Blacks. Furthermore, the association of age and total cholesterol levels with the occurrence of cardiovascular events differed between Blacks and Whites.

**Conclusion:**

The magnitude of associations between risk factors and the presence of atherosclerotic disease differs between race/ethnic groups. These subtle, yet significant differences provide insight in the etiology of cardiovascular disease among race/ethnic groups. These insights aid the race/ethnic-specific implementation of primary prevention.

## Introduction

Cardiovascular disease (CVD) has historically been considered a disease of the developed world.[1] However, CVD is rapidly becoming the largest contributor to morbidity and mortality in growing economies with diverse race/ethnic groups.[2] Furthermore, due to globally increasing mobility large race/ethnic minority groups arise in developed countries. As primary prevention of CVD is key, adequate risk prediction models are necessary to identify and treat high-risk individuals.

To date, most research on primary prevention and risk scores of CVD has been conducted amongst Whites. For example, the landmark Framingham Risk Score (FRS)[3] and the European SCORE[4] have been developed in a largely White population.

Despite recalibrating risk scores for specific race/ethnic groups, it has been shown that existing scores—even scores with ethnicity as a covariate—perform inconsistently among different race/ethnic groups. This results in both under- and overestimating risk, seriously compromising their usefulness in diverse race/ethnic groups.[5] Both QRISK2 and the FRS, identified only 10% to 24% of individuals to be at high risk of those who experienced cardiovascular (CV) events among African Caribbeans. One study recalibrated the FRS and additionally studied the value of the risk factors individually for Whites, Blacks and Mexican Americans; revealing differences in risk factor association with cardiovascular disease.[6] For example, for CVD mortality the hazard ratio (HR) of age was significantly higher in Whites as compared to Blacks and Mexican Americans. Also, the HR for high-density lipoprotein (HDL) cholesterol was significantly higher in Mexican Americans when compared to Whites.

The prevalence of several established cardiovascular risk factors (systolic blood pressure, use of antihypertensive drugs, diabetes, smoking, total cholesterol and HDL-cholesterol)[7] also differs between race/ethnic groups.[8] For example, diabetes is more prevalent in Blacks and Hispanics than in Asians and Whites.[9] But whether differences in absolute risk factor levels also entail race/ethnic differences in the associations with atherosclerosis and CVD has not yet been clarified. This gap in our knowledge has very recently been underlined by the American Heart Association guidelines on the assessment of cardiovascular risk.[10] They strongly recommend “continued research to fill gaps in knowledge regarding short- and long-term atherosclerotic cardiovascular disease risk assessment and outcomes in all race/ethnic groups (…) Further research should include analyses of short- and long-term risk in diverse groups”.

In order to fill this knowledge gap, a large multi-ethnic cohort with a sufficient number of CV events is needed. For this purpose we used the individual participant data meta-analysis USE-IMT cohort.[11] In addition to analyzing the associations with CV events, this cohort also offers the opportunity to assess differences among race/ethnic groups in the association of risk factors to subclinical atherosclerosis measured by mean common carotid intima media thickness (CIMT).

## Methods

### Study population

USE-IMT is an ongoing individual participant data meta-analysis of which the methods have been described in detail elsewhere.[11] In short, general population cohorts were identified using literature search and expert suggestions. For inclusion in USE-IMT, cohorts were required to have available baseline data on age, sex, blood pressure, cholesterol fractions, smoking status, use of antihypertensive medication, diabetes mellitus and CIMT-measurements and follow-up information on occurrence of cardiovascular events. For the current analysis, we included the participating cohorts of which data on ethnicity on an individual level were available (n = 9). Cohorts that did not have information on ethnicity were included if it was reasonable to assume that >95% of the participants belonged to one race/ethnic group due to either selection of participants or race/ethnic homogeneity of the source population (n = 6). Ethnicity was recoded when applicable, to create uniform race/ethnic groups for analysis. Details concerning this recoding process and the original classification can be found in S1 Table. As our research is focused on the first-time events in asymptomatics, only individuals to whom the Framingham criteria [7] are applicable were included in the current analyses.

### Ethics statement

This study was approved by the institutional review committee of the University Medical Centre Utrecht. Each individual cohort obtained approval from a local Ethical Review Board and written informed consent from all participants. All authors exchanged a material transfer agreement. This study conforms to the declaration of Helsinki.

### USE-IMT, CIMT and CV events

Out of the 17 cohorts participating in USE-IMT, we included 15 cohorts[12–26], consisting of 66,213 individuals. After excluding individuals of whom individual ethnicity was not known (n = 285) or who had already experienced a cardiovascular event (n = 5,717) 60,211 individuals were included for analyses. This group consisted of 46,788 Whites, 7,200 Blacks, 3,816 Asians and 2,407 Hispanics.

Incomplete data on mean common CIMT, cardiovascular risk factors, and (time to) CV events, approximately 12% of total values, were imputed, as described previously.[11] This imputation was done to increase power and to reduce omitted variable bias.[27]

Average mean common CIMT was calculated for each individual using the maximum set of carotid angles, near and/or far wall measurements, and left and/or right side measurements that were assessed within each cohort. Time to first fatal or non-fatal myocardial infarction or stroke (hemorrhagic or ischemic) was used as a primary endpoint in this analysis.

### Statistical analysis

Baseline data are represented as means with standard deviations (SD) for continuous variables and as percentages for categorical variables. We describe our population both by their original cohort (Table 1) and by race/ethnic group (Table 2). All statistical analyses were performed in R (version 2.15.1).

**Table 1:** Details of participating USE-IMT cohorts.

Cohort	Country	Individuals (n)	White (n)	Black (n)	Asian (n)	Hispanic (n)	Age (years)	Gender (% men)	Mean CIMT (mm, sd)	FU (years)	Stroke (n)	MI (n)	CV event (n)
ARIC[12]	USA	14,728	10,750	3,978	-	-	54.0	43.1	0.65 (0.15)	12.3	540	829	1,285
CAPS[13]	Germany	4,798	4,798	-	-	-	49.4	48.1	0.72 (0.14)	8.0	91	68	153
CHS[14]	USA	4,365	3,700	661	4	-	72.4	38.6	0.87 (0.16)	10.4	645	590	1,108
CIRCS[15]	Japan	1,939	-	-	1,939	-	65.5	75.7	-	7.9	89	23	109
EAS[16]	UK	980	980	-	-	-	68.9	49.1	0.77 (0.28)	12.2	28	11	39
FATE[17]	Canada	1,560	1,548	10	1	1	49.4	99.8	0.72 (0.18)	7.5	11	22	33
Hoorn[18]	NLD	308	308	-	-	-	68.7	48.1	0.85 (0.15)	7.4	6	11	16
KHID[19]	Finland	908	908	-	-	-	51.2	100.0	0.76 (0.16)	13.1	58	114	159
Malmö[20]	Sweden	5,163	5,163	-	-	-	57.5	40.5	0.77 (0.15)	10.4	184	186	251
MESA[21]	USA	6,814	2,622	1,893	803	1,496	62.2	47.2	0.76 (0.18)	6.0	116	140	355
NBS[22]	NLD	1,172	1,160	9	3	-	60.8	46.7	0.83 (0.11)	3.8	3	13	16
NOMAS[23]	USA	1,494	256	295	33	910	68.9	40.0	0.73 (0.09)	7.9	63	55	108
OSACA2[24]	Japan	484	-	-	484	-	65.5	49.6	0.87 (0.27)	4.4	22	2	24
Tromsø[25]	Norway	5,699	5,699	-	-	-	59.3	47.2	0.78 (0.16)	10.1	352	534	830
Whitehall[26]	UK	9,799	8,896	354	549	-	61.2	67.1	0.78 (0.15)	6.0	110	138	244
**Combined**		**60,211**	**46,788**	**7,200**	**3,816**	**2,407**	**59.0**	**51.3**	**0.75 (0.17)**	**9.1**	**2,318**	**2,736**	**4,730**

Abbreviations. ARIC: Atherosclerosis Risk in Communities Study; CAPS: Carotid Atherosclerosis Progression Study; CHS: Cardiovascular Health Study; CIRCS: Circulatory Risk in Communities Study; EAS: Edinburgh Artery Study; FATE: The Firefighters and Their Endothelium Study; Hoorn: The Hoorn Study; KIHD: Kuopio Ischaemic Heart Disease Risk Factor Study; Malmö: Malmö Diet and Cancer Study, MESA; Multi-race/ethnic Study of Atherosclerosis; NBS: Nijmegen Biomedical Study 2; NOMAS: Northern Manhattan Study; OSACA2: Osaka Follow-Up Study for Carotid Atherosclerosis 2; Tromsø: Tromsø Study; Whitehall: Whitehall II Study; CIMT: mean common carotid intima media thickness; FU: follow-up duration; MI: myocardial infarction; USA: United States of America; UK: United Kingdom; NLD: The Netherlands.

**Table 2:** Baseline properties per race/ethnic group.

	Whites	Blacks	Asians	Hispanics	Total
Individuals (n)	46,788	7,200	3,816	2,407	60,211
Age, years	58.4 (10.1)	58.6 (9.9)	64.3 (7.3)	63.4 (10.0)	59.0 (10.0)
Gender, % men	52.4	39.3	64.5	44.5	51.3
CIMT, mm	0.74 (0.17)	0.74 (0.18)	0.78 (0.20)	0.74 (0.15)	0.75 (0.17)
Smoking, % yes	19.6	23.7	21.3	13.8	20.0
Diabetes, % yes	5.9	18.0	8.2	17.0	7.9
BMI, kg/m^2^	26.6 (4.4)	29.6 (6.0)	23.7 (3.3)	29.0 (5.0)	26.9 (4.8)
TC, mmol/L	5.8 (1.2)	5.4 (1.1)	5.3 (0.9)	5.2 (1.0)	5.7 (1.2)
HDL, mmol/L	1.4 (0.4)	1.4 (0.4)	1.4 (0.4)	1.2 (0.3)	1.4 (0.4)
LDL, mmol/L	3.8 (1.1)	3.4 (1.0)	3.2 (0.8)	3.2 (0.9)	3.7 (1.1)
TG, mmol/L	1.5 (1.0)	1.2 (0.8)	1.5 (0.9)	1.7 (1.0)	1.5 (0.9)
SBP, mmHg	130 (21)	131 (22)	136 (21)	132 (22)	131 (21)
DBP, mmHg	77 (12)	78 (12)	79 (12)	76 (12)	77 (12)
Stroke events (n)	1,732	398	125	63	2,318
MI events (n)	2,302	328	46	60	2,736
CV events (n^a^)	3,780	665	168	117	4,730
Mean FU duration (years)	9.3	9.6	6.8	6.6	9.1
10-y event rate^b^	8.1	9.2	6.7	7.8	8.2

All data represent means (sd), unless stated otherwise.

^a^ Number of individuals with a CV event (first-time stroke or MI).

^b^10-year event rate, estimated using Kaplan-Meier analysis.

To examine the influence of ethnicity on associations of risk factors with mean common CIMT and CV events, we used linear regression models and Cox regression models, respectively. For both questions, we first fitted a model containing the Framingham risk factors (age, sex, systolic blood pressure (SBP), HDL-cholesterol, total cholesterol, current smoking, presence of diabetes and antihypertensive drug use)[7] and ethnicity as independent variables. Mean common CIMT (linear regression-log transformed) and a combined endpoint of first-time stroke or myocardial infarction (Cox regression) were used as an outcome, respectively. To take between-study-variation into account, we took a random effects approach for each original cohort. We then extended this model by adding the interaction terms for ethnicity with each risk factor. Whites were the reference group in all analyses.

We first compared the interaction model to the first model using a likelihood ratio test. Subsequently, we tested the significance of the interaction terms. Finally, we calculated hazard ratios and betas (with 95% confidence intervals) for each risk factor in each race/ethnic group, and tested for significance of individual interaction terms using a Wald test. Due to the large number of tests performed, we took a conservative threshold for interaction at p_interaction_ = 0.05 (commonly accepted p-values for interactions are 0.1 or 0.2). In these analyses, Whites were the reference group. Interaction terms were only considered significant when significantly adding to the model without interaction terms and when significantly different from Whites.

No comparisons between ethnicities were performed, except for the comparisons to Whites. A two-sided significance level of 0.05 was used.

## Results

The White race/ethnic group was derived from 13 cohorts that originated from Canada, the United States of America (USA) and Europe. The Black group was derived from 7 cohorts, with the majority of individuals residing in the USA. The Asian group comprised of 8 cohorts. The majority of Asians (63.5%) originated from Japan; another substantial part (21% of total) of Asians was Chinese American. The Hispanics were derived from three (two American and one Canadian) cohorts. The characteristics of each cohort are described in Table 1. The mean age for the entire cohort was 59 years (SD 10), 51% were male. Median follow-up time was 9 years, with a total follow-up of 547,887 person years. During this time 4,730 CV events, i.e., fatal or non-fatal myocardial infarction or stroke, occurred.

Asians and Hispanics were older than Blacks and Whites (64 versus 58 years). While sex in Whites was balanced, Blacks and Hispanics were mainly women (61% and 55%) and Asians were predominantly male (65%). Blacks and Hispanics had notably higher BMIs and a higher prevalence of diabetes mellitus as compared to Whites and Asians. Hispanics had lower rates of smoking (13.8%) compared to Blacks (23.7%), while the prevalence of smoking in Whites and Asians was comparable (respectively 19.6% and 21.3%). Mean LDL-cholesterol was highest in Whites (3.8 mmol/L). In Asians and Hispanics mean LDL-cholesterol was 3.2 mmol/L, in Blacks it was 3.4 mmol/L. Systolic blood pressure was 136 mmHg in Asians, 132 mmHg in Hispanics, 131 mmHg in Blacks and 130 mmHg in Whites. Baseline data for each race/ethnic group are presented in Table 2.

### Common CIMT

Mean common CIMT was 0.74 mm in Blacks, Whites and Hispanics and 0.78 mm in Asians.

The model with risk factors and interaction terms with ethnicity fitted the data better than the model with risk factors alone (likelihood ratio test p<0.001). The interaction terms of ethnicity with age, HDL-cholesterol, smoking and systolic blood pressure were statistically significant (p <0.05).

We thus found that for specific ethnicities risk factor associations were significantly different from Whites.

**Fig 1:**
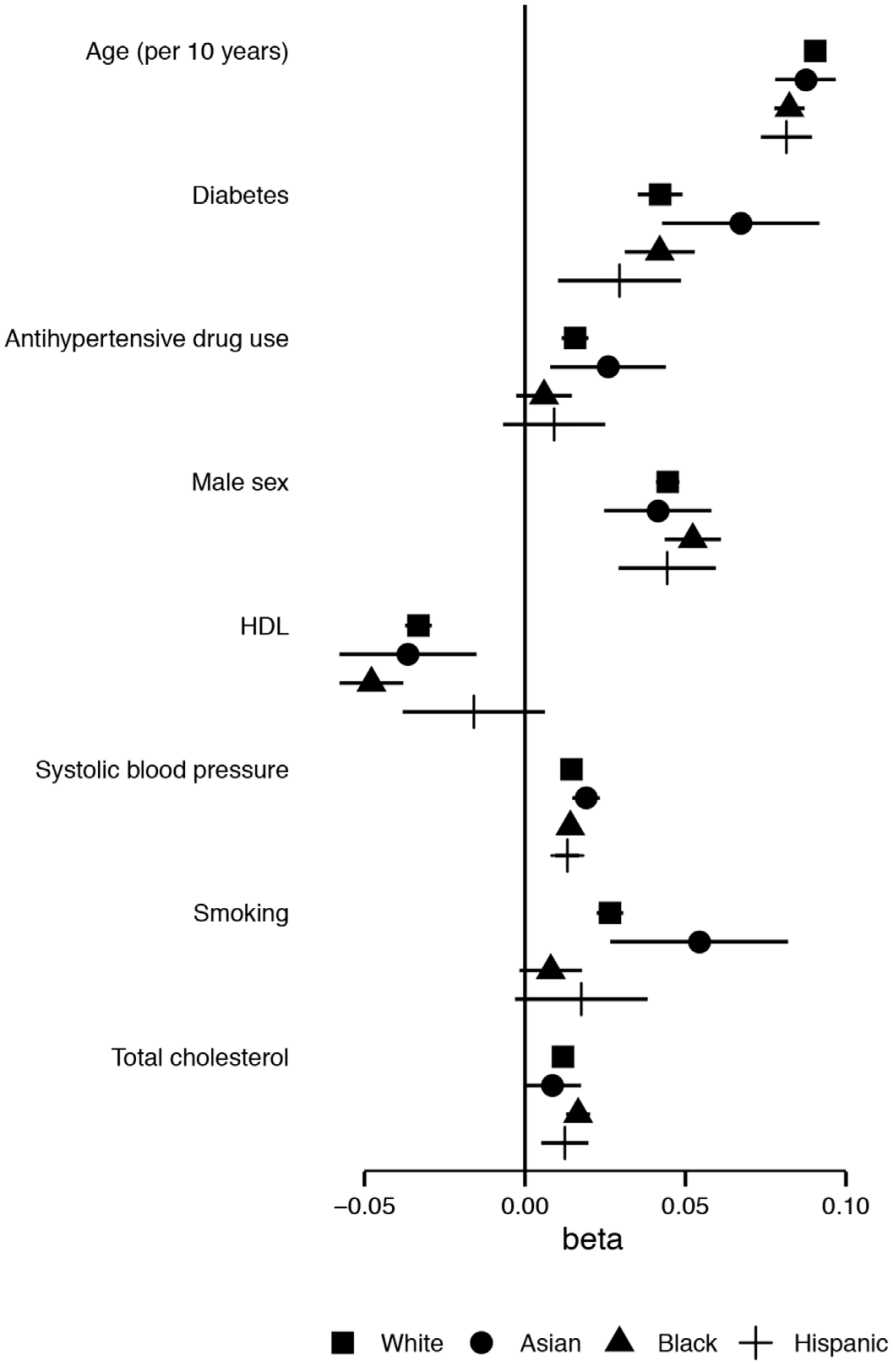
Association between risk factors and mean common CIMT, by ethnicity. Point estimates for betas, lines represent 95% confidence intervals.

The magnitude of the association (i.e. betas of the linear regression model) of age with logCIMT was significantly smaller in Blacks and Hispanics as compared to Whites: 0.08/10 years (91% of the White beta) and 0.09/10 years (97% of the White beta), respectively. This means that every 10-year increase in age gives less increase in mean common CIMT in Blacks and Hispanics than it does in Whites. The beta for HDL-cholesterol per 1 mmol/L increase was -0.05 (144%) in Blacks (a stronger inverse association) as compared to the beta in Whites. The beta for smoking (yes vs. no) was 0.01 (30% of the beta in Whites). In Asians the beta for SBP was 0.02 (131% of the White beta). All betas are plotted in Fig 1 and are presented in Table 3.

**Table 3:** Effects of the Framingham risk factors in race/ethnic groups for the outcomes log CIMT (betas) and CV events (hazard ratios).

	White	Reference	Black	% difference	Asian	% difference	Hispanic	% difference
**IMT (betas, 95% CI)**								
**Age**	0.09 (0.09–0.09)	100%	0.08 (0.08–0.09)^a^	91%	0.09 (0.08–0.10)	97%	0.08 (0.07–0.09)^a^	90%
Diabetes	0.04 (0.04–0.05)	100%	0.04 (0.03–0.05)	100%	0.07 (0.04–0.09)	160%	0.03 (0.01–0.05)	70%
BP drug use	0.02 (0.01–0.02)	100%	0.01 (-0.00–0.01)	38%	0.03 (0.01–0.04)	166%	0.01 (-0.01–0.03)	58%
Gender	0.04 (0.04–0.05)	100%	0.05 (0.04–0.06)	118%	0.04 (0.02–0.06)	93%	0.04 (0.03–0.06)	100%
**HDL**	−0.03 (−0.04−−0.03)	100%	−0.05 (−0.06−−0.04)^a^	144%	−0.04 (−0.06−−0.02)	110%	−0.02 (−0.04−0.01)	48%
**SBP**	0.01 (0.01–0.02)	100%	0.01 (0.01–0.02)	98%	0.02 (0.01–0.02)^a^	131%	0.01 (0.01–0.02)	91%
**Smoking**	0.03 (0.02–0.03)	100%	0.01 (-0.00–0.02)^a^	30%	0.05 (0.03–0.08)	205%	0.02 (-0.00–0.04)	66%
TC	0.01 (0.01–0.01)	100%	0.02 (0.01–0.02)	140%	0.01 (-0.00–0.02)	73%	0.01 (0.01–0.02)	105%
**CV events** ^b^ **(HRs, 95% CI)**								
**Age**	1.89 (1.86–1.93)	100%	1.52 (1.44–1.60)^a^	80%	1.75 (1.49–2.01)	92%	1.69 (1.48–1.90)	89%
Diabetes	1.95 (1.86–2.05)	100%	2.13 (1.97–2.30)	109%	2.62 (2.20–3.03)	134%	2.59 (2.20–2.98)	133%
BP drug use	1.28 (1.21–1.35)	100%	1.50 (1.34–1.66)	117%	1.47 (1.14–1.79)	115%	0.93 (0.53–1.32)	72%
Gender	1.52 (1.45–1.59)	100%	1.35 (1.19–1.51)	89%	1.82 (1.42–2.22)	119%	1.56 (1.17–1.95)	102%
HDL	0.67 (0.58–0.76)	100%	0.64 (0.44–0.85)	96%	0.88 (0.46–1.29)	132%	0.91 (0.33–1.49)	137%
SBP	1.15 (1.14–1.17)	100%	1.18 (1.15–1.21)	103%	1.12 (1.05–1.20)	98%	1.20 (1.12–1.29)	104%
Smoking	1.96 (1.89–2.04)	100%	1.70 (1.53–1.87)	87%	1.59 (1.25–1.93)	81%	1.58 (1.09–2.08)	81%
**TC**	1.09 (1.07–1.12)	100%	1.20 (1.13–1.26)^a^	110%	0.95 (0.78–1.13)	87%	1.15 (0.96–1.34)	105%

The top half of the table displays betas and 95% confidence intervals of the Framingham risk factors for log CIMT for each race/ethnic group. The bottom half of the table displays hazard ratios and 95% confidence intervals of the Framingham risk factors for CV events for each race/ethnic group. The % difference columns express the percentage difference in effect size (beta or hazard ratio) as compared to the White race/ethnic group. Risk factors printed in bold have significantly different effect sizes among race/ethnic groups (significant interaction).

^a^ Indicates significant difference as compared to Whites (p<0.05)

^b^ Cardiovascular events (first-time stroke or MI).

### CV events

The 10-year event rate was 6.7% in Asians, 7.8% in Hispanics, 8.1% in Whites and 9.2% in Blacks.

**Fig 2:**
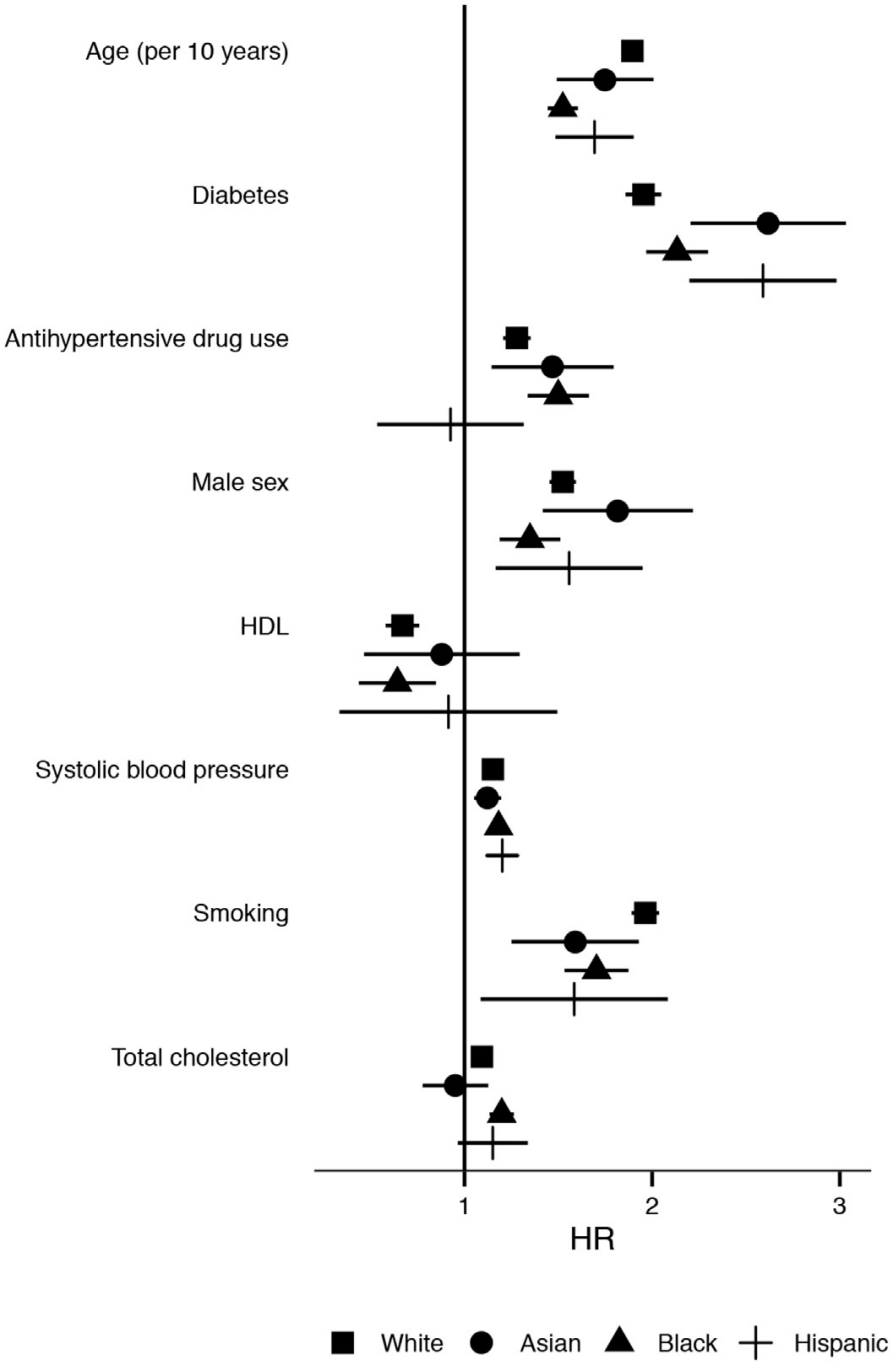
Association between risk factors and first-time stroke or myocardial infarction, by ethnicity. Point estimates for hazard ratio’s, lines represent 95% confidence intervals.

The model including the risk factors and the interaction terms with ethnicity fitted the data better than the model without interaction terms (likelihood ratio test p <0.001). Both age and total cholesterol had a significant interaction with ethnicity. The HR for CV events (first-time stroke or MI) per 10-year increase in age was lower in Blacks than in Whites (1.52 (1.44–1.60), 80% of the HR in Whites). The HR for a 1 mmol/L increase of total cholesterol was higher in Blacks (1.20 (1.13–1.26), 140%) compared to Whites. The hazard ratios of the Framingham risk factors per ethnic group are plotted in Fig 2.

## Discussion

Our study shows that the associations of Framingham risk factors with subclinical atherosclerosis and CV events have similar directions across race/ethnic groups. However, the magnitude of the associations differs significantly for several risk factors among race/ethnic groups.

### Prevalence of risk factors

The prevalence of risk factors in our cohort was unevenly distributed among the race/ethnic groups (Table 2). Race/ethnic differences in the prevalence of CVD risk factors have been previously described. Similar to our study, smoking was least prevalent in Asians.[28] Also diabetes was more prevalent in Blacks and Hispanics than in Asians and Whites[9], and the higher prevalence of diabetes was accompanied with higher BMI in these groups.[29] Also in accordance with literature, TC and LDL-cholesterol levels were highest in Whites, HDL-cholesterol levels were lowest in Hispanics and triglyceride levels were lowest in Blacks.[30]

However, in contrast with these similarities between our data and published literature, blood pressure was highest in Asians, which is opposite to the findings in the Asia Pacific cohort.[31] Possibly, this incongruence between our study and Asia Pacific can be explained by differences in age, as the Asians in our cohort are older than the Whites (in the Asia Pacific cohort the group from Australia and New Zealand is older than the Asians).

The observations made above indicate that our cohort is fairly similar to other cohorts described in literature, thereby suggesting that our results might be generalizable.

### CIMT and CVD—Differences and similarities

For Blacks, an increase in age was related to both an increase in mean common CIMT and a higher risk of CV events, in our analyses. The magnitude of these associations, however, was significantly smaller than in Whites. Although the direction of the association of age is the same in Blacks as in Whites, an increasing age has less effect on disease in Blacks than in Whites. This indicates that age is one of the factors that should be should weighted when developing a prediction model specifically for Blacks.

HDL-cholesterol and smoking in Blacks, SBP in Asians and age in Hispanics showed a different magnitude of the association with mean common CIMT as compared to Whites. However, these differences in the magnitude with mean common CIMT were not detected in the analysis of CV events. This might be due to the difference in power between linear regression and Cox proportional hazards analysis (which is driven by the number of events), or a difference in biology of CIMT and the actual occurrence of CV events.

The association between TC and CV event was significantly higher for Blacks as compared to Whites. The coefficient for TC on mean common CIMT was also higher than the coefficient in Whites, although this difference did not reach statistical significance.

When it comes to geographical differences, some of these differences have been reported before. The Asia Pacific study reported lower HRs for triglycerides and SBP for respectively coronary heart disease and hemorrhagic stroke, when comparing Asian countries with Australia and New-Zealand.[32] The INTERHEART study[33] showed in an analysis of 52 countries across the globe that odds ratios for myocardial infarction were comparable, although (small) differences in effect sizes exist. These geographical differences are in agreement with our race/ethnicity-specific findings.

Race/ethnicity-specific risk prediction should be exercised with discretion. Unwanted medical “discrimination” that could be perceived by patients should be avoided at all costs. On the other hand optimal risk estimation is of paramount importance for medical care. Adding race/ethnicity to risk prediction equations thus should be implemented with care and reasons to do so should be clarified to all parties, as to avoid undesirable situations in health care and society.

### Limitations

Due to differences in ascertaining and recording ethnicity per cohort, misclassification might have occurred and may have diluted our results to some extent. While we are aware of the growing number of people from mixed backgrounds, people of mixed race/ethnicity were excluded from the current analyses. Also, our Asian and Hispanic race/ethnic groups were relatively small, which may have led to a lack of power. Therefore, our conclusions on these groups should be interpreted with caution.

We were unable to take immigration status or acculturation into account, although time since immigration has been shown to influence mean common CIMT, risk factors levels and risk of cardiovascular events.[34,35] Therefore we cannot determine in which way immigration status might influence our results, or whether the results are influenced at all.

In our CV events analysis we were unable to take differences in prevention and treatment strategies into account, these might differ among the ethnic groups and influence the occurrence of CV events.

## Conclusion

In conclusion, the associations of the Framingham risk factors with atherosclerosis (CIMT) and CVD had similar directions across race/ethnic groups. However, the magnitude of associations between risk factors and the presence of atherosclerotic disease differs between race/ethnic groups. These subtle, yet significant differences provide insight in the etiology of cardiovascular disease among race/ethnic groups. These insights aid the race/ethnic-specific implementation of primary prevention.

## Supporting Information

S1 Table Recoding of race/ethnicity per cohort. (PDF)
