# Supplementary material for: Race/Ethnic Differences in the Associations of the Framingham Risk Factors with Carotid IMT and Cardiovascular Events
Source: PLoS One. 2015 Jul 2;10(7):e0132321. doi: 10.1371/journal.pone.0132321 (PMC4489855; doi:10.1371/journal.pone.0132321)
Supplement: S1 Table — (PDF) [file pone.0132321.s001.pdf]

S1 Table. Recoding of race/ethnicity per cohort

Cohort name	Original classification	Recoded for analysis
ARIC	Caucasian	Caucasian
	Black	Black
CAPS	All Caucasian	Caucasian
CHS	Caucasian	Caucasian
	Black	Black
	American Indian	Other
	Asian or Pacific Islander	Asian
	Other	Other
CIRCS	All Japanese[a]	Asian
EAS	All Caucasian[a]	Caucasian
FATE	Caucasian	Caucasian
	Black	Black
	Hispanic	Hispanic
	Asian or Pacific Islander	Asian
	Canaduab	Other
	East Indian	Asian
	Other	Other
Hoorn	All Caucasian[a]	Caucasian
KIHD	All Caucasian[a]	Caucasian
Malmö	All Caucasian[a]	Caucasian
MESA	Caucasian	Caucasian
	Chinese American	Asian
	Black	Black
	Hispanic	Hispanic
NBS	Canada	Caucasian
	Surinam	Black
	Austria	Caucasian
	Belgium	Caucasian
	Hungary	Caucasian
	Morocco	Other
	Iraq	Other
	Finland	Caucasian
	Greece	Caucasian
	Libya	Other
	United States	Caucasian
	Luxembourg	Caucasian
	Indonesia	Asian
	Germany	Caucasian
	Netherlands	Caucasian
	Spain	Caucasian
	Great Britain	Caucasian
	Turkey	Other
	Yugoslavia	Caucasian
	Mexico	Hispanic
	Netherlands Antilles	Black
	Poland	Caucasian
	Russia	Caucasian
	Japan	Asian
	Uruguay	Other
	Italy	Caucasian
	New Guinea	Other
	Netherlands Indie	Other
NOMAS	Caucasian	Caucasian
	Chinese American	Asian
	Black	Black
	Hispanic	Hispanic
OSACA2	Japanese	Japanese
Tromsø	All Caucasian[a]	Caucasian
Whitehall	Caucasian	Caucasian
	South Asian	Asian
	Black	Black
	Other	Other

[a] Ethnicity was not available on an individual level, but it was reasonably assumable that the vast majority of the individuals in the cohort have this ethnicity.

Individuals coded with 'other' ethnicity were excluded from the analysis.
